# Pulmonary Manifestations in Systemic Sclerosis: Hospital-Based Descriptive Study

**DOI:** 10.7759/cureus.8649

**Published:** 2020-06-16

**Authors:** Ravindrachari Mulkoju, Vinod Kumar Saka, Manju Rajaram, Rashmi Kumari, Vir S Negi, Madhusmita Mohanty Mohapatra, Vishnukanth Govindaraj, Dharm Prakash Dwivedi, Vemuri Mahesh Babu

**Affiliations:** 1 Pulmonary Medicine, Jawaharlal Institute of Postgraduate Medical Education and Research, Puducherry, IND; 2 Dermatology, Jawaharlal Institute of Post Graduate Medical Education and Research, Puducherry, IND; 3 Internal Medicine: Rheumatology, Jawaharlal Institute of Postgraduate Medical Education and Research, Puducherry, IND; 4 Internal Medicine, Jawaharlal Institute of Postgraduate Medical Education and Research, Puducherry, IND

**Keywords:** interstitial lung disease, systemic sclerosis, pulmonary involvement, nailfold capillaroscopy, high resolution computed tomography, modified rodnan scoring, carbon monoxide diffusion capacity

## Abstract

Introduction

Prevalence of systemic sclerosis (SSc)-related organ injury is difficult to estimate as it occurs early in SSc, even though patients are often asymptomatic. As the patients with organ damage have a poor prognosis, all the patients should be carefully evaluated and followed‑up in the initial periods. This facilitates the early identification and initiation of appropriate therapy. This study emphasizes on different clinical manifestations and early predictors of lung involvement by using clinical, radiological, and pulmonary function tests in a tertiary care centre.

Materials and methods

A total of 53 SSc cases, who satisfied American College of Rheumatology (ACR) 2013 criteria, without any overlap syndromes were included in the study. All patients underwent thorough clinical examination along with Modified Rodnan Scoring (MRS) assessment, nailfold capillaroscopy (NFC), chest X-ray (CXR), HRCT thorax, 2D-echocardiography, spirometry and diffusion lung study by carbon monoxide (DLco).

Results

Out of 53 patients, four were male and 49 were female. Twenty-one patients had limited SSc (lcSSc) and 32 had diffuse SSc (dcSSc). Eighty-three per cent of subjects presented with skin manifestations and 34% with respiratory complaints. Reticulonodular opacities and ground glassing were the predominant radiological abnormalities suggestive of non-specific interstitial pneumonia (NSIP) followed by usual interstitial pneumonia (UIP). Pulmonary hypertension was predominant in patients with lcSSc. Thirty-eight patients had a restrictive pattern of spirometry. Forty-four patients showed deranged DLco, among which two patients showed an isolated decrease in DLco. Thirty-seven patients had abnormal NFC among which dropout pattern was predominant. MRS was significantly correlated with pulmonary involvement by DLco and HRCT.

Conclusions

SSc can affect the lungs even before developing obvious clinical pulmonary manifestations. DLco and HRCT play a critical role in detecting early lung involvement and predicting the outcomes in SSc. Higher modified Rodnan’s score, which has a significant correlation with DLco and HRCT can be used to predict early visceral involvement in resource-limited settings.

## Introduction

Systemic sclerosis (SSc) or scleroderma is a type of collagen vascular disorder manifested by fibroblast and endothelial cell dysfunctions. Changes in fibroblast and endothelial cell function result in collagen overproduction and immune system abnormalities. These changes in the different systems lead to collagen overproduction, multi-organ damage and death [1]. Apart from collagen overproduction, systemic sclerosis patients may also exhibit proliferative, obliterative and inflammatory changes in the vascular system and other target organs. The prevalence of systemic SSc-related organ injury is challenging to estimate because it occurs early in SSc, such as pulmonary fibrosis in the first two years and renal crisis in the first four years of disease onset, even though patients are often asymptomatic [1,2]. As patients with organ damage have a poor prognosis, all patients should be carefully evaluated and monitored via follow-up in the initial periods for organ involvement to facilitate the early identification and initiation of appropriate therapy. To our knowledge, studies on pulmonary involvement in SSc are limited in India [3]. This study emphasizes different clinical manifestations and early predictors of lung involvement in SSc to decrease morbidity and mortality.

## Materials and methods

Objectives

The objective of this study was to evaluate the pulmonary manifestations by clinical, radiological, and pulmonary function tests as well as to assess the early predictors of lung involvement in SSc.

Study design

This was a descriptive cross-sectional study conducted in the Department of Pulmonary Medicine in collaboration with the Department of Clinical Immunology and Department of Dermatology and sexually transmitted diseases in a tertiary care teaching institute in south India. This observational study was carried out from April 2014 to 2016, during which 53 patients were enrolled. The study protocol was approved by the Institute Scientific Advisory Committee and the Institute Ethics Committee. Data collection was prospective and informed, and written consent was obtained from relevant groups.

Selection and description of participants

Study participants included all SSc patients age 18 years and older who satisfied the American College of Rheumatology 2013 criteria [4]. Patients with active infections, a history of previous pulmonary diseases such as tuberculosis, malignancies with a risk of radiation exposure to the thorax, and overlap with other collagen vascular diseases or autoimmune diseases were excluded from the study.

A detailed clinical history was taken for all patients with respect to all systemic complaints. Patients underwent a thorough clinical examination along with a modified Rodnan skin score (MRS) assessment, chest X-ray (CXR), high-resolution computed tomography (HRCT) of the thorax, two-dimensional (2D) echocardiography, spirometry, diffusion lung study by carbon monoxide (DLco), and nailfold capillaroscopy (NFC).

Technical information

All patients underwent CXR and HRCT of the thorax. In X-ray, we observed pulmonary opacities, including distribution of different patterns such as consolidation, micronodules, reticulations, and volume loss. We also looked for pulmonary hypertension by defining dilatation of central pulmonary artery diameter >15 mm in women and >16 mm in men [5]. HRCT was performed in the dorsal decubitus position, sequential mode with helical equipment, 1.0-mm thick slices, 10.0-mm intervals, and reconstructed with hard filter. Both pulmonary parenchymal and mediastinal windows, without contrast administration, were obtained. The study review was performed by the resident physician and radiodiagnosis specialist of the hospital. Computed tomography (CT) findings were noted as reticular opacities, ground-glass opacities, ground-glass opacities associated with reticular opacities, reticulonodular opacities, honeycombing, pleural alterations observing the site, distribution, and extent of involvement, as well as the presence of mediastinal lymphadenopathy (nodes of size >10 mm in their smallest diameter). CT diagnosis of pulmonary hypertension was made based on enlarged pulmonary artery diameter >29 mm, which is greater than the ascending aorta at the same level [6].

We used 2D-echocardiography in all patients to rule out structural and functional abnormalities. Tricuspid regurgitation pressure gradient >40 mmHg (tricuspid regurgitation velocity >3.2 m/s) with an assumed right atrial pressure of 10 mmHg (thus corresponding to a systolic pulmonary artery pressure >50 mmHg) was taken as the cutoff value for the diagnosis of pulmonary hypertension [7].

Spirometry and DLco were conducted using a Jaeger MasterScreen CareFusion (Germany) pulmonary function test machine, in accordance with the American Thoracic Society (ATS)/European Respiratory Society (ERS) task force lung function testing standardization guidelines. The degree of restriction and reduction in the diffusing capacity was assigned as per the 2005 ATS/ERS Task Force lung function guidelines [8]. The spirometry classification of severity of obstruction was made in accordance with the 2013 Global Initiative for Chronic Obstructive Lung Disease guidelines [9].

The severity of skin involvement was determined per MRS in which skin thickening is assessed by palpation of the skin in 17 areas of the body (fingers, hands, forearms, arms, feet, legs and thighs, face, chest, and abdomen) using a 0 to 3 scale, where 0 = normal, 1 = mild thickness, 2 = moderate thickness, and 3 = severe thickness. Total skin score can range from 0 (no thickening) to 51 (severe thickening in all 17 areas). Individuals with skin thickening above the elbows, knees, and on the chest were classified as diffuse cutaneous SSc (dcSSc). Individuals with skin thickening restricted to the face, forearms, hands, and fingers were classified as limited cutaneous SSc (lcSSc), also known as calcinosis, Raynaud's phenomenon, esophageal dysmotility, sclerodactyly, and telangiectasia (CREST) syndrome.

Pulmonary involvement was defined as either structural abnormality by radiology (CXR/HRCT) or functional abnormality by spirometry/DLco. All diagnosed cases of SSc were given standard recommended treatment.

Statistical analysis

Statistical analysis was conducted with STATA 2012 software (StataCorp, LLC, College Station, TX). The distribution of data for categorical and ordinal variables, such as gender of patients, signs, and symptoms, and radiological parameters, were expressed as frequencies and percentages. A comparison of variables between the groups was carried out using the chi-square test or Fisher's exact test. Data for continuous variables such as age, biochemical parameters, and pulmonary function parameters were expressed as mean with standard deviation or median with range. A comparison of variables between the groups was carried out using independent Student's t-test or Mann-Whitney U test, whichever was appropriate. All statistical analyses were carried out at a 5% level of significance, and a p-value <0.05 was considered significant.

## Results

Sixty-five patients with features of SSc were screened, and 53 patients who satisfied the inclusion criteria were enrolled in the study. Of those 53 patients, four patients were men, and 49 patients were women. Ages of the study population ranged from 20 years to 55 years, with a mean of 37.4 ± 9.6 years. The mean age of onset was 35.2 ± 9.0 years, with a minimum age of onset being 18 years and the maximum age being 51 years. The median duration of disease was two years, with an interquartile range of one to three years. In our study, of 53 SSc patients, 21 patients had lcSSc (CREST syndrome), and 32 patients had dcSSc.

**Figure 1:**
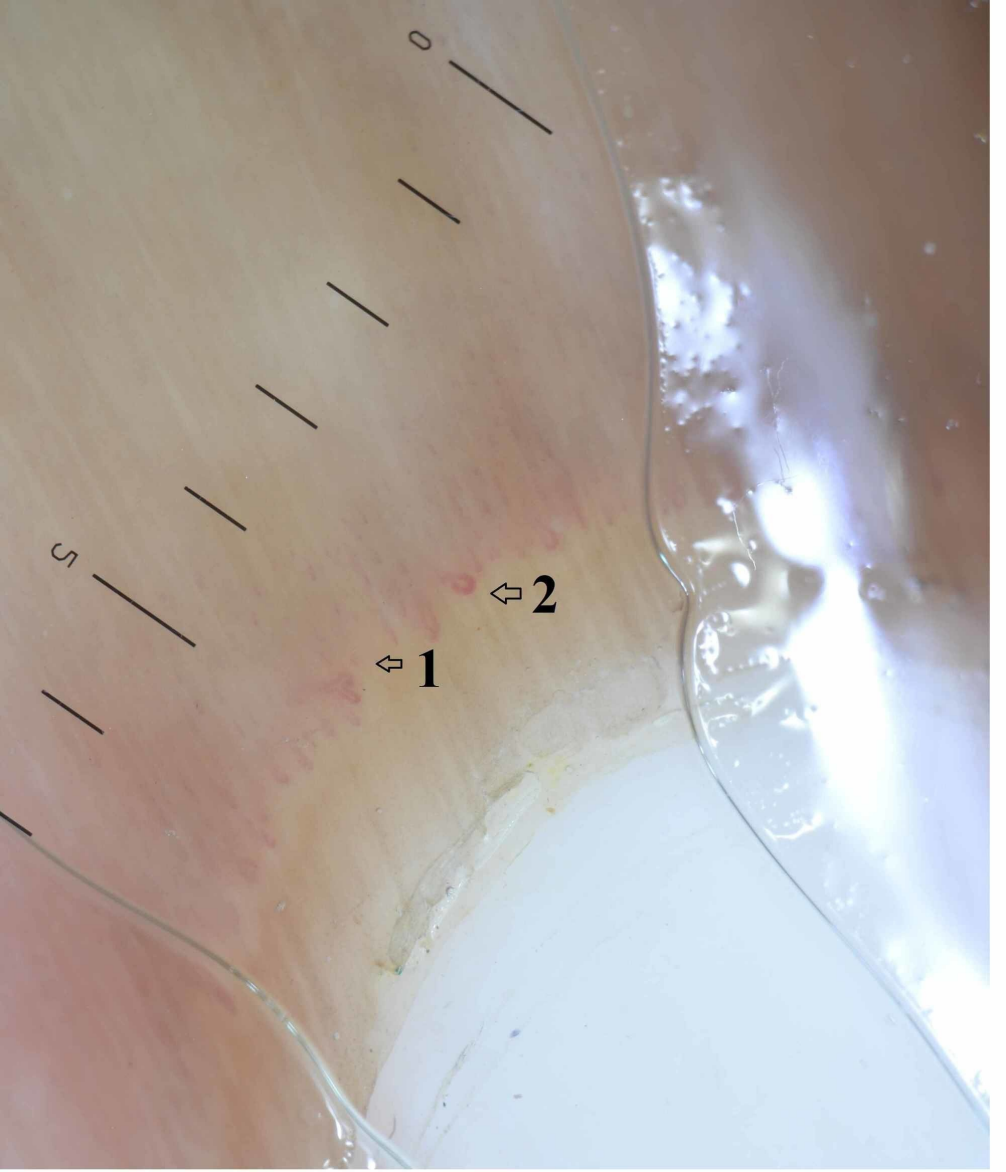
Nailfold capillaroscopy in systemic sclerosis Nailfold capillaroscopy in systemic sclerosis showing (1) dropout/loss of capillaries and (2) dilated and tortuous capillaries

The clinical manifestations of study patients are tabulated in Table 1. Across all patients, skin manifestations were the most common presentation, and dyspnea was the predominant respiratory symptom. Different patterns of abnormal capillary arrangements in NFC were identified in 37 patients using dermatoscopy. Of those 37 patients, dropouts/loss of capillaries was the most common pattern observed in 19 patients, followed by a combined pattern of dropout with hemorrhages in six patients, and hemorrhages alone in five patients. Dropout with hemorrhage and tortuosities was seen in three patients, tortuous capillaries in two patients, dropout with tortuous capillaries in one patient, and hemorrhage with tortuous capillaries in one patient (Figure 1).

**Table 1:** Clinical manifestations observed in systemic sclerosis

Characteristics	Number (N = 53)	Percent (%)
Dyspnea	18	34.0
Chest pain	2	3.8
Sclerodactyly	44	83.0
Raynaud's phenomenon	44	83.0
Skin pigmentation	44	83.0
Inadequate mouth opening	23	43.4
Neck sign	8	15.1
Calcinosis	1	1.9
Dysphagia	1	1.9
Modified Rodnan scoring (>16)	30	56

Radiological abnormalities (CXR and HRCT) were separately observed in lcSSc and dcSSc. Normal CXR was seen in 18 (34%) patients and HRCT in 12 (23%) patients. Abnormal patterns are tabulated in Table 2.

**Table 2:** Radiological findings in patients with systemic sclerosis HRCT, high-resolution computed tomography; PAH, pulmonary arterial hypertension *Parenchymal opacities mixed with pleural alterations

	Limited Systemic Sclerosis (N = 21)		Diffuse Systemic Sclerosis (N = 32)	
Chest X-ray Finding	Frequency	Percent (%)	Frequency	Percent (%)
Normal	10	48	8	25
Reticulonodular opacities	3	14.3	10	32
Reticular opacities	4	19	8	25
Dilated pulmonary artery	2	9.5	1	3
Emphysema	1	4.8	1	3
Others	1	4.8	4	12
HRCT Finding				
Normal	6	28.5	6	19
Ground-glass opacity	6	28.5	12	37.5
Subpleural cysts/ honeycombing	6	28.5	11	34
Interlobular septal thickening	5	24	11	34
Dilated pulmonary artery (PAH)	5	24	3	9
Traction bronchiectasis	5	24	2	6
Others*	2	9.5	9	28

Pulmonary function test (spirometry) was categorized into obstructive, restrictive, and mixed patterns. DLco was performed in 52 patients. One patient could not perform the test because of inadequate mouth opening. Severity among each category was graded according to ATS 2011 classification criteria and is tabulated in Table 3. 

**Table 3:** Spirometry and diffusion lung study by carbon monoxide observations in systemic sclerosis patients SD, standard deviation; DLco, diffusion lung study by carbon monoxide

Characteristics	Number Mean ± SD (N = 53)	Percent (%)
Spirometry involvement	42	81
Obstructive pattern	2	4
Restrictive pattern		
Mild	20	38
Moderate	16	30
Severe	2	4
Mixed pattern	2	4
DLco involvement	44	83
Mild (61-79% of predicted)	16	30.2
Moderate (41-60% of predicted)	23	43.4
Severe (20-41% of predicted)	5	9.4
Very severe (<20% of predicted)	0	0

Right atrial and ventricular dilatations and tricuspid regurgitation pressure gradient were measured by 2D-echocardiography in all 53 patients. Eleven patients with SSc satisfied pulmonary hypertension criteria by echocardiography. Of those 11 patients, six (54.5%) patients belonged to the limited variant and five (45.5%) patients belonged to the diffuse variant.

In our study, patients were assessed for skin thickness using MRS. Among all 53 patients, the mean MRS was 15.34 ± 6.82. A cutoff score of 16 was taken to classify patients with mild (MRS <16) and severe (MRS >16) skin sclerosis. Of 53 patients, 30 patients had severe skin thickness, and 23 patients had mild skin thickness. Among 23 patients with mild skin thickness, 12 (52%) patients had lcSSc, and 11 (48%) had dcSSc. Among 30 patients with severe skin thickness, eight (27%) patients had lcSSc, and 22 (73%) had dcSSc.

## Discussion

SSc-related mortality is attributed to end-stage lung disease, either pulmonary fibrosis or pulmonary arterial hypertension. At autopsy, approximately 90% of SSc patients had evidence of lung involvement. The present study was designed to evaluate different pulmonary manifestations of SSc by clinical, radiological, and pulmonary function tests and their role in diagnosing the disease, even though patients were clinically asymptomatic, to detect organ involvement in the initial stages. This discussion is chiefly focused on the features observed in the present study.

SSc and clinical manifestations

Despite the heterogeneity, <2% of patients show skin involvement, which is critical in the initial diagnosis and classification. In lcSSc, skin involvement is insidious, confined to the extremities/face while in dcSSc, skin involvement is very rapid, extending beyond the extremities/face. Cutaneous manifestations are often associated with or preceded by Raynaud's phenomenon (episodic, cold-induced, or stress-induced digital ischemia) of fingers with or without arthralgias. Vascular manifestations, such as pulmonary arterial hypertension, are typically more common in lcSSc. Widespread internal organ involvement and mortality are more common in dcSSc due to severe cardiac, pulmonary, gastrointestinal, and renal involvement. In both subsets, ischemic digital ulcers can occur earlier because of local ischemia and vascular insufficiency. Though pathophysiology is unclear, calcinosis (i.e., abnormal dystrophic calcium deposition) in soft tissues such as digits, elbows, and knees independent of serum calcium and phosphorous levels-causing pain, local inflammation, atrophy, ulceration, and joint contractures leading to disabilities of the involved parts-can occur in approximately 25% of SSc patients [10].

In our study, 83% of patients presented with skin manifestations such as sclerodactyly, Raynaud's phenomenon, and skin pigmentation. Respiratory complaints such as dyspnea were seen in 34% of patients, suggesting skin manifestations are more common and present early in SSc.

SSc and NFC

Raynaud's phenomenon is an important diagnostic feature for most autoimmune rheumatic diseases. In normal conditions or primary Raynaud's phenomenon, nailbed shows regular deposition of capillaries during capillaroscopic examination. On the other hand, in secondary Raynaud's phenomenon (secondary to rheumatic diseases), there will be one or more alterations of the capillaroscopic pattern of nailfold capillaries, which should alert the physician to the possibility of an underlying connective tissue disease not yet detected. Evaluation of NFC in autoimmune rheumatic diseases might represent a tool for the prediction of microvascular organ involvement by considering the systemic microvascular derangement at the capillary nailfold. In this scenario, in 2001, LeRoy and Medsger proposed criteria for the early diagnosis of SSc, which include a combination of clinical (Raynaud's phenomenon), imaging (NFC), and laboratory (presence of SSc-specific autoantibodies) data considering the importance of NFC [11]. Scleroderma patterns usually characterized by the presence of architectural disorganization, giant capillaries, hemorrhages, loss of capillaries (dropouts), angiogenesis, and avascular areas characterize >95% of SSc patients [12].

We observed an abnormal capillary pattern in 37 SSc patients. The predominant pattern observed was the loss of capillaries (dropouts; 19 patients, 35.8%), which is classified as an active form of capillaroscopic pattern [11]. NFC pattern was correlated with the involvement of the lungs by the disease. We observed that pulmonary involvement was seen in 92% (34 patients) of abnormal NFC, and 87.5% (14 patients) with normal NFC suggested there was an association between risk of lung injury and NFC pattern [13].

SSc and pulmonary function testing

Survival in SSc has been inversely correlated to the degree of restrictive ventilatory defect on pulmonary function tests. All patients with SSc should be screened for the development of interstitial lung disease (ILD) and pulmonary hypertension at the time of diagnosis and periodically thereafter. Pulmonary function testing is an essential, readily available, noninvasive means to detect SSc-related pulmonary complications in early stages. For patients with minimal to no restriction, the 10-year survival was 87%, compared to a survival rate of 75% and 58% in patients with moderate and severe restriction, respectively [14]. Functional vital capacity and DLco are both identified as adverse prognostic markers in SSc-related lung injury. DLco is reduced in almost all patients with other pulmonary function test abnormalities, and a reduced DLco is the single most significant marker of poor outcome and correlates with the extent of lung disease on HRCT.

Impaired DLco in SSc-induced lung injury can usually be secondary to two main pathological conditions, namely ILD and pulmonary hypertension. In regular clinical practice, abnormally low DLco can be found even in the absence of these complications. Reduced DLco levels in ILD are due to a reduction in alveolar volume and/or thickening of the alveolar-capillary membrane. Reduced DLco levels in pulmonary hypertension are due to vascular remodeling, which leads to vessel wall tightening and arterial stiffness. In SSc patients, the presence of baseline isolated marked reduction in DLco (<55% of predicted) might characterize a peculiar SSc subset that may precede the development of pulmonary hypertension, and progression of pulmonary vascular disease can be linked to decreasing DLco trends [15].

The present study showed that in the majority of patients (n = 44, 85%) with abnormal DLco, spirometry was abnormal in 42 (95.5%) patients. Two patients had isolated impairment in DLco, which can be seen in patients with SSc with normal spirometry without any clinical, radiological, or echocardiographic evidence of lung involvement [16]. This result reinforces the need for careful clinical follow-up of patients with isolated DLco reduction, even in clinically asymptomatic patients, and that DLco is preferable to spirometry in detecting lung involvement such as ILDs.

SSc and pulmonary hypertension

SSc patients have the highest prevalence of pulmonary hypertension among all patients with collagen vascular diseases. Pulmonary hypertension can occur in all forms of SSc and is associated with early mortality. Isolated pulmonary hypertension is now the most common cause of disease-related death in lcSSc, the most common variant of scleroderma [16]. Steen and colleagues suggested that small vessel vasculopathy is the main pathophysiology of pulmonary hypertension [16]. This would be consistent with declining DLco, which often predates the onset of pulmonary hypertension. Another study also suggested a correlation between the NFC dropout pattern in the nailfold beds and the presence of isolated pulmonary hypertension in limited scleroderma [17].

In our study, two patients (4%) had isolated pulmonary hypertension, which was evidenced by normal spirometry and impairment in DLco with abnormal NFC pattern. We, therefore, hypothesize that progressive pulmonary capillary dropout and/or dysfunction might underlie the pathogenesis of this late-stage complication.

Transthoracic echocardiography is one of the most common diagnostic modalities used to screen for pulmonary hypertension in SSc and has a sensitivity of 90% [18]. The diagnosis of pulmonary hypertension widely varies with echocardiography characteristics and the cutoff limits used. Some studies proved the correlation between echocardiographic measurements of pulmonary arterial hypertension and right heart catheterization (RHC) values. Between 55% and 86% of patients with an echocardiography finding suggestive of pulmonary hypertension (right ventricular systolic pressure [RVSP] 30 to 40 mmHg or higher with or without symptoms) will have pulmonary hypertension on RHC. When the measurement of RVSP is combined with increased right atrial or right ventricular size, reduced pulmonary artery acceleration, and decreased right ventricular function, the specificity of echocardiography for the diagnosis of pulmonary hypertension will be increased. Though RHC is the gold standard investigation for diagnosing pulmonary hypertension, it was not done in our study; 54.5% (six patients) of lcSSc and 45.5% (five patients) of dcSSc showed pulmonary hypertension by echocardiography.

CXR is the least sensitive investigation to diagnose pulmonary hypertension but has high specificity (up to 100%) [19]. Though sensitivity is less for pulmonary hypertension, based on dilatation of central pulmonary artery diameter, loss of peripheral vasculature, and right ventricular filling of the retrosternal space on the lateral image can predict pulmonary hypertension. Predictive findings on HRCT are the mean pulmonary artery diameter and the ratio of the mean pulmonary artery diameter to the ascending aorta diameter [6]. Based on these criteria, CXR diagnosed pulmonary hypertension in three (5.6%) patients (two in lcSSc and one in dcSSc), HRCT in eight (15%) patients (five in lcSSc and three in dcSSc), and echo in 11 (21%) patients (six in lcSSc and five in dcSSc), suggesting 2D-echocardiography is more sensitive in diagnosing pulmonary hypertension and is more common in lcSSc than dcSSc.

SSc-induced pulmonary hypertension usually occurs after 10 to 15 years and is common in the lcSSc variant. In contrast, ILD is common in dcSSc and usually occurs in the initial five years after diagnosis. However, patients with limited or diffuse disease can present at any stage with pulmonary hypertension, whether associated or not with ILD. Patients with isolated pulmonary hypertension without ILD are classified into Group 1 (pulmonary hypertension), while patients of pulmonary hypertension with ILD are classified into Group 3, of the pulmonary hypertension classification [20].

SSc and chest radiology

ILD and pulmonary hypertension are the two most common pulmonary manifestations of SSc. Two-thirds of SSc patients develop scleroderma ILD, and approximately 20% of SSc patients develop pulmonary hypertension, which is usually associated with severe lung injury, although it can be an isolated manifestation. Other roles of imaging in scleroderma are to identify patients who are likely to respond to medicines, to assess treatment efficacy, and to exclude other associated cardiac and esophageal abnormalities.

Evidence of pulmonary disease has been described in CXRs in 20% to 65% of patients affected by SSc. Chest radiographs can be normal in early lung involvement and even in some patients with pulmonary symptoms. Abnormal radiographic findings are observed in two-thirds of symptomatic patients, whereas only 25% to 44% of patients with fibrosis may show subtle radiographic abnormalities [21]. HRCT has been shown to be more sensitive than CXR in diagnosing and characterizing SSc-related lung diseases, and findings on CT correlate more closely with pulmonary function test abnormalities. In this scenario, HRCT is now well-established as a simple noninvasive and sensitive investigation to detect and characterize ILDs. SSc-related lung diseases manifest as increased ground-glass opacity, low lung volumes, and interstitial reticular thickening predominates in the basal areas of lungs. Ground glass opacity is the most common finding on HRCT; however, it is an isolated finding in a minor proportion of cases. In the late stages of lung involvement, pulmonary fibrosis manifests as traction bronchiectasis and honeycombing changes. Fibrosis can present in 55% to 65% of patients with SSc and up to 96% of those with abnormal pulmonary function test results [22].

Additionally, HRCT correlates well with results of pulmonary function tests, demonstrating that SSc-related lung injury is a restrictive disorder associated with low lung volumes and diffusion disorder, which impairs diffusion capacity for carbon monoxide [23]. Apart from diagnosing and characterizing the nature and severity of ILD, HRCT can be used to predict the outcome of SSc-related ILD. The absence of lung involvement in CT at the time of disease presentation is a good long-term prognostic indicator of SSc-ILD. In this context, HRCT findings, in combination with pulmonary function test results, will improve the prediction of diagnostic accuracy and prognostic certainty.

SSc and ILD

Early autopsy studies suggested that up to 100% of patients of SSc will have parenchymal involvement in the form of ILD [6,7]. ILD most commonly complicates dcSSc, but it can also be associated with lcSSc or with SSc without cutaneous involvement (SSc sine scleroderma) in 40% of cases [24]. As many as 90% of patients will have interstitial abnormalities on HRCT, and 40% to 75% will have changes in pulmonary function tests [25]. Despite high sensitivity, HRCT can be normal in some patients who have pulmonary function test abnormalities and in some patients who have abnormal chest auscultation (i.e., crackles). These patients showed abnormal HRCT findings during follow-up scans. Despite these limitations, the presence of a normal HRCT at baseline predicts a low likelihood for the development of SSc-ILD, as 85% of these patients still have a normal HRCT at a mean follow-up of five years [26]. Because of the above reasons, the diagnosis of SSc-ILD is frequently made by combining clinical findings, pulmonary function tests, and HRCT abnormalities. In most cases, lung biopsies are rarely required for ruling out other associated parenchymal processes.

A common HRCT pattern seen in SSc-ILD is a greater proportion of ground-glass opacities with a lower degree of reticulation suggestive of nonspecific interstitial pneumonia (NSIP) [27]. In up to two-thirds of patients, even with treatment, ground-glass opacities progress to fibrosis and lead to honeycombing/traction bronchiectasis and/or bronchiectasis formation over time. Honeycomb cysts, a marker for usual interstitial pneumonia (UIP) and pulmonary fibrosis, can be seen in up to one-third of patients with SSc-ILD and are more common in patients with lcSSc, suggesting that patients with SSc-ILD disease may have a mixture (or overlap) of UIP and NSIP patterns. The pattern of HRCT findings has a good correlation with histology. Ground glass opacities/consolidation correlate with active inflammation, and reticular opacities/honeycombing correlate with fibrotic lesions. Patients with CT features of ground-glass opacities respond better to treatment, as they are markers of inflammation and reversible lung injury [28]. According to Desai et al. and other autopsy studies, SSc-ILD histopathological findings can show a combination of pulmonary fibrosis and inflammation, suggesting SSc can be associated with a mixture (or overlap) of UIP and NSIP patterns [27].

In our study, HRCT detected ILD in 41 (77%) of 53 patients (81% of dcSSc and 71% of lcSSc) among which ground-glass opacities (i.e., NSIP) was the most common ILD in both forms. We defined pulmonary involvement as either radiological (CXR/HRCT of the thorax) or echocardiographic or pulmonary function test (spirometry/DLco) abnormalities. Forty-four patients (83%) were identified as having pulmonary involvement based on these criteria. Thirty-seven patients (85%) who identified as having pulmonary involvement had skin involvement either in the form of Raynaud's phenomenon, skin pigmentation, or sclerodactyly. Among 44 patients with pulmonary involvement, 24 were asymptomatic (no cough/dyspnea/chest pain) for respiratory complaints, of which 18 patients had DLco impairment, and 16 patients had parenchymal abnormalities on HRCT. None of the respiratory complaints were significantly associated with lung involvement (p>0.05).

As skin findings were common in patients with SSc, MRS scoring was correlated with different investigations used to define pulmonary involvement. Baseline MRS ≥20 was associated with heart and lung involvement and predicted mortality in the subsequent four years [29]. We correlated pulmonary involvement by using skin thickness with different methods of assessments (spirometry, DLco, HRCT of the thorax, 2D-echocardiography). Our observation suggested MRS was significantly correlated with pulmonary involvement by HRCT as well as DLco (p < 0.05).

Limitations

As this is a cross-sectional observational study, lung involvement was studied at one point. Further follow-up with a large study population is needed to monitor the progression of disease and treatment response to plan better management protocol.

## Conclusions

SSc can affect the lungs even before developing obvious clinical pulmonary manifestations. The activity of the disease is better correlated with DLco and HRCT; therefore, these two tests play a critical role in detecting early lung involvement and predicting outcomes in SSc. Higher MRS, which has a significant correlation with DLco and HRCT, can be used to predict early visceral involvement in resource-limited settings.
